# Treatment with mitochondrial targeting antibiotics improves survival outcomes after Flock House virus infection in young and aged Drosophila melanogaster

**DOI:** 10.21203/rs.3.rs-6816306/v1

**Published:** 2025-06-10

**Authors:** Dean Bunnell, Madelyn Buhl, Justin McGee, Grace Milas, Stanislava Chtarbanova

**Affiliations:** University of Alabama; University of Alabama; University of Alabama; University of Alabama; University of Alabama

**Keywords:** Drosophila melanogaster, innate immunity, aging, virus infection, antibiotics, mitochondrial unfolded protein response

## Abstract

Aged organisms are more susceptible to infectious diseases, including infections with RNA viruses. Mitochondrial dysfunction is one of many hallmarks of aging that could affect this increased susceptibility, as the relationship between immunity and metabolism is crucial to manage infections. Using *Drosophila melanogaster*- Flock House virus (FHV) host-virus interactions model system, previous work has identified differences in young and aged flies’ ability to modulate oxygen consumption rates (OCR). Here, we hypothesized that interventions that reduce OCR could improve survival of FHV, as observed in young flies. Tetracycline (TTC) and rifampicin (RIF) antibiotics disrupt mitochondrial translation and transcription respectively because of mitochondria’s bacterial ancestry. The mitochondrial unfolded protein response (UPR^mt^) is activated by mitochondrial stressors, including reactive oxygen species, defects in oxidative phosphorylation, and mitonuclear protein imbalance. UPR^mt^ activation initiates retrograde signaling to the nucleus, prompting transcription, translation, and import of nuclear proteins to resolve stress. We showed TTC or RIF treatment extended survival in young and aged flies after FHV infection, independently of virus load modulation. Furthermore, we demonstrate that bacterial loads are not significantly different between FHV-infected flies and controls, and that the protective effect of TTC likely occurs independently of its antimicrobial properties. We observed increased expression of genes involved in the UPR^mt^, glycolysis, and oxidative stress response with TTC treatment. Our results suggest perturbing mitonuclear protein balance with TTC or RIF could activate the UPR^mt^ and improve outcomes of virus infection.

## Introduction

Living organisms are constantly exposed to stressors capable of altering physiological functions that may have implications on their survival. These stressors include but are not limited to exposure to variable environments, nutrient limitations, injury, oxidative stress, and disease states [1]. Cells, tissues, and organisms possess mechanisms of ‘adaptive homeostasis’ [2] capable of detecting these constant and variable stressors and generating the appropriate response to restore homeostasis and in many cases promote survival. A key component of this response is the ability to employ these adaptive mechanisms quickly and inactivate the response when the stress or damage has been resolved. An abundance of evidence in many model organisms has demonstrated that aging results in a functional decline in adaptive homeostatic mechanisms [3]. The immune system also experiences a progressive functional decline with age, or immunosenescence [4]. Ultimately, this decline renders organisms more susceptible to infections, including RNA virus infections [5–8]. A major gap exists in our understanding of the underlying factors implicated in older individuals’ increased susceptibility to infections. The world population of people 65 years or older is projected to more than double by 2050 [9], so identifying strategies to promote aged organisms’ ability to survive infection and improve health will be critical in the near future.

*Drosophila melanogaster* and FHV provide a powerful aged host-RNA virus interaction model to elucidate mechanisms that improve the aged host’s survival. Although invertebrates such as *Drosophila* lack the adaptive immunity found in vertebrates, they provide a unique model to investigate innate immunity. In vertebrates, innate immunity is the first line of defense when faced with infection and aids adaptive immune system activation, as the two branches must act synergistically to fight infection. FHV is an insect virus with a positive sense, RNA genome composed of two single-stranded RNA molecules copackaged in a virion. Its genetic simplicity has made it a widely used model in host-virus interactions, and its replication cycle is well characterized [10]. It replicates within replication spherules formed on the outer mitochondrial membrane [11–13]. FHV infection is pathogenic to flies and results in mortality within days [12, 14–16].

During pathogenic infections, two evolutionarily conserved defense strategies must act in conjunction to limit pathology. Resistance involves detection of the pathogen by recognition of pathogen associated molecular patterns (PAMPs) or damage associated molecular patterns (DAMPs) followed by activation of effector mechanisms capable of reducing pathogen load. while disease tolerance limits tissue damage and consequently disease severity without affecting pathogen load. In *Drosophila*, several antiviral resistance mechanisms have been identified in response to FHV infection, including RNA interference (RNAi) [15], apoptosis [17], and hemocyte-mediated phagocytosis [18]. Disease tolerance mechanisms have not been characterized as extensively as resistance mechanisms. The epigenetic regulator, Histone H3 lysine 9 (H3K9) methyltransferase G9-alpha, has been shown to play a role in disease tolerance to FHV by preventing hyperactivation of the JAK-STAT pathway [11].

Our lab previously showed that FHV infection causes mortality in aged flies’ significantly faster than in young flies without observing an increase in viral titers [14], suggesting an age-related decline in disease tolerance to FHV. Transcriptomic analysis revealed many genes whose products are involved in metabolic processes, mitochondrial structure, and mitochondrial respiration were regulated to a stronger extent in older flies 48h p.i. [14]. This finding implicated host metabolism as a potential factor differentially regulated with age that may increase susceptibility to FHV. Previously, we conducted a longitudinal, single-fly respirometry study to measure whole organismal oxygen consumption rates (OCR) in single flies as a proxy for metabolic rate and mitochondrial function. In this study, we determined if metabolic rates differed with age after FHV infection and whether differences may be correlated with mortality [19]. We found young flies reduced OCR after FHV infection, but aged flies showed no significant changes in OCR, providing more evidence that metabolism could impact age-related susceptibility to FHV [19]. In fact, the reduction of OCR in young flies is indicative of an evolutionarily conserved strategy of metabolic rate depression or hypometabolism, which has been reported as a survival strategy in the presence of various stressors across organisms (reviewed in [20, 21]), including the promotion of disease tolerance [22].

Several classes of antibiotics have demonstrated protective effects in the context of viral infections, cancer, sepsis, and inflammatory disorders that are not attributed to direct antimicrobial effects [23–27]. Since mitochondria retain many characteristics of their bacterial ancestors, many classes of antibiotics can act via similar mechanisms on eukaryotic mitochondria and cause dysfunction [28, 29]. In bacteria, tetracycline inhibits bacterial protein synthesis by occupying the A-site of the bacterial 30S ribosomal subunit and blocking recruitment of the aminoacyl-tRNA [30]. Rifampicin inhibits bacterial DNA transcription by inhibiting RNA polymerase [31]. In eukaryotes like *Drosophila*, these antibiotics alter the balance of nuclear-encoded and mitochondrial-encoded proteins, which is crucial for mitochondrial electron transport chain function. High-resolution respirometry has shown doxycycline, a tetracycline class antibiotic, decreases OCR in flies [29], and *Drosophila’s* mitochondrial RNA polymerase was shown to be inhibited by RIF [32]. In *Drosophila* S2 cells, rifampicin was shown to have an antiviral effect against *Drosophila* C Virus (DCV) and Cricket Paralysis Virus (CrPV), which was associated with increased expression of detoxification enzymes of the cytochrome P450 family [33]. *Drosophila* ND23 is a nuclear gene that encodes a highly conserved subunit of the mitochondrial ETC Complex I. Previous studies have shown that *ND23*^*60114*^ mutants display abnormal mitochondrial morphology, reduced ATP levels and have a significantly shorter lifespan in comparison to wild type controls [34]. Additionally, *ND23*^*60114*^ mutants show increased expression of *Hsp22*[35], a chaperone protein that has been identified to play a role in the *Drosophila* UPR^mt^ [36].

Modest perturbation of the mitochondrial electron transport chain (ETC) function has been shown to increase lifespan in *Drosophila, C*. elegans, and mice [37–39]. Additionally, the mitochondrial unfolded protein response (UPR^mt^) can be activated by various stressors including mitonuclear protein imbalance, misfolded protein accumulation, reactive oxygen species (ROS), mtDNA depletion, or mitochondrial toxins, and involves retrograde signaling to the nucleus to mount a transcriptional response to relieve mitochondrial stress [40–43]. This includes the transcription of genes that encode for mitochondrial chaperones, proteases, antioxidants, and glycolytic genes, all of which function to alleviate mitochondrial stress (reviewed in [44]). UPR^mt^ activation has been shown to improve disease tolerance mechanisms in mammalian studies [24, 25]. Therefore, many studies indicate that the presence of mitochondrial stress and subsequent activation of the UPR^mt^ represents a hormetic response, where a mild amount of stress can have beneficial effects [24, 25, 45, 46].

In this study, we sought to investigate whether antibiotics that impair mitochondrial metabolism improve survival of FHV infection with aging. We observed that TTC and RIF extended survival of FHV infection in both young and older flies without reducing virus. Survival extension occurred independently of antimicrobial properties after TTC treatment. In virus infected flies of both age cohorts, we found that TTC treatment affected the expression of genes implicated in the UPR^mt^, glycolysis, and oxidative stress response. Additionally, in comparison to *wild type* controls, *ND23*^*60114*^ mutants displayed extended end point survival when infected with FHV. Overall, our findings indicate that mitochondrial dysfunction and resulting UPR^mt^ activation improves survival outcomes of FHV infection likely by functioning as part of a disease tolerance mechanism.

## Materials and Methods

### Drosophila stocks, handling and aging

*Oregon R* stock (#2376) was obtained from Bloomington *Drosophila* Stock Center (Bloomington, IN, USA). Flies were maintained in vials containing Nutri-Fly Bloomington formulation food (Genesee Scientific, Cat #: 66–113) in a 25°C incubator with controlled 12 h:12 h light:dark cycle. For experimentation, *Oregon R* male flies were collected, aged, and flipped every 3–4 days into vials of fresh food until reaching the desired age. Young and aged cohorts were 5–9 days-old and 28–32 days-old, respectively.

*ND23* ^*60114*^ stock was kindly provided by Dr. Barry Ganetzky and *Canton S* (#64349) was obtained from Bloomington *Drosophila* Stock Center (Bloomington, IN, USA). Heterozygous controls were obtained by crossing *Canton S* males with *ND23*^*60114*^ virgin females (Control: n = 53, FHV: n = 47) and the reciprocal cross (Control: n = 49, FHV = 56). Flies were aged to 5–9 days in vials containing Nutri-Fly Molasses formulation food (Genesee Scientific, Cat #: 66–116) in a 25°C incubator with controlled 12 h:12 h light:dark cycle. All fly lines were confirmed *Wolbachia*-negative prior to experimentation.

#### Virus stock and injections

FHV stock was propagated by infecting low passaged Schneider’s *Drosophila* line 1 (DL-1) cells. Virus stock solution was stored at − 80°C. All injections were conducted with a Nanoject II or III (Drummond Scientific), as described previously [14]. Flies were individually injected at the appropriate age with either 59.8 nL of cell culture media (Schneider’s media, 10% fetal bovine serum, and 1:100 penicillin-streptomycin (pen/strep U/mL); control injection) or FHV (2.57× 10^5^ 50% Tissue Culture Infective Dose (TCID_50_)/mL). The control-injected cohort was injected prior to the FHV-injected cohort to prevent cross-contamination. Flies were anesthetized with CO_2_ on a fly pad (Genesee Scientific) and manipulated with a paintbrush during injections. Injected flies of the same experimental condition were then placed in groups of 10–15 flies per vial and allowed to rest at room temperature for 1 hour after injection before being placed in a 22°C incubator. For survival assays, survival status was recorded daily, and flies were flipped into vials of fresh food every other day.

#### Antibiotic Food Preparation

Nutri-Fly Bloomington formulation food (Genesee Scientific, Cat #: 66–113) or Corn-syrup/Soy media (Archon Scientific, W1) was prepared per manufacturer’s directions and supplemented with the appropriate dose of antibiotic or equal volume of vehicle serving as a control for each antibiotic treatment. Tetracycline (ThermoFisher Scientific, Alfa Aesar, Cat#: J61714) was supplemented to the food at the final concentrations of 0.05 mg/mL [47], 0.005 mg/mL, and 0.0005 mg/mL. The tetracycline stock solution was prepared using 80% ethanol, which served as the vehicle. Rifampicin (Tokyo Chemical Industry Co., Ltd., Product#: R0079) was supplemented to the food at a final concentration of 500 mg/L [48]. The rifampicin stock solution was prepared using DMSO, which served as the vehicle. Flies were placed on vehicle- or antibiotic-supplemented food only after they have been injected with either cell culture media (control) or FHV.

#### Reverse transcription quantitative real time PCR (RT-qPCR)

Total RNA was extracted from 5 whole flies (24, 72, or 120h p.i.) using the Quick-RNA MiniPrep kit (Zymo Research) and cDNA was prepared from 500 ng total RNA using the High-Capacity cDNA Reverse Transcription kit (Applied Biosystems). Triplicate cDNA samples were amplified using the PowerTrack^™^ SYBR Green Master Mix (Applied Biosystems) with a StepOnePlus Real time PCR system according to the manufacturers’ protocols. Primers for all genes analyzed can be found in **Table S1**. The ΔΔCt method was used to analyze the expression of UPR^mt^, metabolic, and oxidative stress response genes. The expression of each gene of interest was normalized to expression of *ribosomal protein L32 (RpL32)* and to expression of control-injected flies to observe changes resulting from TTC treatment. The ΔCt method was used to analyze the expression of *FHV RNA1* for virus load and copy number of bacterial *16S rRNA* for bacterial load. Each value was normalized to expression of *ribosomal protein L32 (RpL32)*.

### Bacterial load assay and real time quantitative PCR (qPCR) for 16S rRNA gene

Young and aged *Oregon R* males were injected with 59.8 nL of either cell culture media (control) or FHV 5 days post injection (d p.i.), 10 whole flies were collected and frozen per sample. To facilitate DNA extraction from gram-positive bacteria, samples were homogenized in 1,200 uL of Enzymatic Lysis Buffer with Lysozyme for 1 hr at 37 ° C and centrifuged for 2 minutes at 16,000 g and supernatant removed [49]. Then, the Wizard Genomic DNA Purification kit’s (Promega, A1120) protocol for Isolation of Genomic DNA from Gram Positive and Gram-Negative Bacteria was followed. DNA concentrations were determined using a NanoDrop One Spectrophotometer. The StepOnePlus Real time PCR system was utilized for all qPCR reactions. Each reaction in a 96-well plate contained 1 uL diluted DNA and 9 uL SYBR Green-containing Mastermix (Applied Biosystems). Three technical replicates were run for each sample and each set of primers. 8FM and Bact515R were the primers used to amplify the bacterial *16S rRNA* gene. *RpL32* forward and *RpL32* reverse primers were used to amplify *RpL32*. Primer sequences may be found in **Table S1**. The thermal cycling protocol for amplification was 50°C for 2 minutes, denaturation for 10 minutes at 95°C, then 40 cycles of 30 seconds at 95°C, 1 minute at 59°C and 30 seconds at 72°C. Gene levels of *16S rRNA* were normalized to *RpL32*.

#### Generation of axenic flies

Male and female *Oregon R* flies were placed in an egg laying chamber and allowed to lay eggs overnight onto apple juice plates with a smear of yeast paste. Sterile water and paintbrush were used to collect eggs from the plate. Working within a biological safety cabinet, collected eggs were placed in 10% bleach for 5 minutes for dechorionation. Next, the eggs were washed 3 times in 70% ethanol, followed by 3 washes in sterile water. Eggs were collected and transferred to autoclaved, NutriFly Bloomington formulation food. Validation of treatment was performed by extracting DNA from individual flies [50] and subsequently used for PCR with universal bacterial *16S rRNA* primers (listed in **Table S1**) [51] and by plating a single fly homogenate on LB agar plates and placed at 37°C overnight to monitor bacterial colony formation (**Fig. S1**). Male flies were only collected from vials that confirmed successful axenic treatment 4 days post-eclosion and aged to 5–9d for injections.

#### Statistical analysis

Statistical analyses were performed with GraphPad Prism software (version 10.4.0) for MAC. The Log-rank (Mantel-Cox) test was used to compare survival curves. Significance of gene expression, virus load, and bacterial load was determined by two-way ANOVA test followed by a Tukey’s multiple comparisons test. For all comparisons, *p* < 0.05 was considered significant.

## Results

### Tetracycline and rifampicin extend survival of FHV infection without reducing virus load

**Figure 1:**
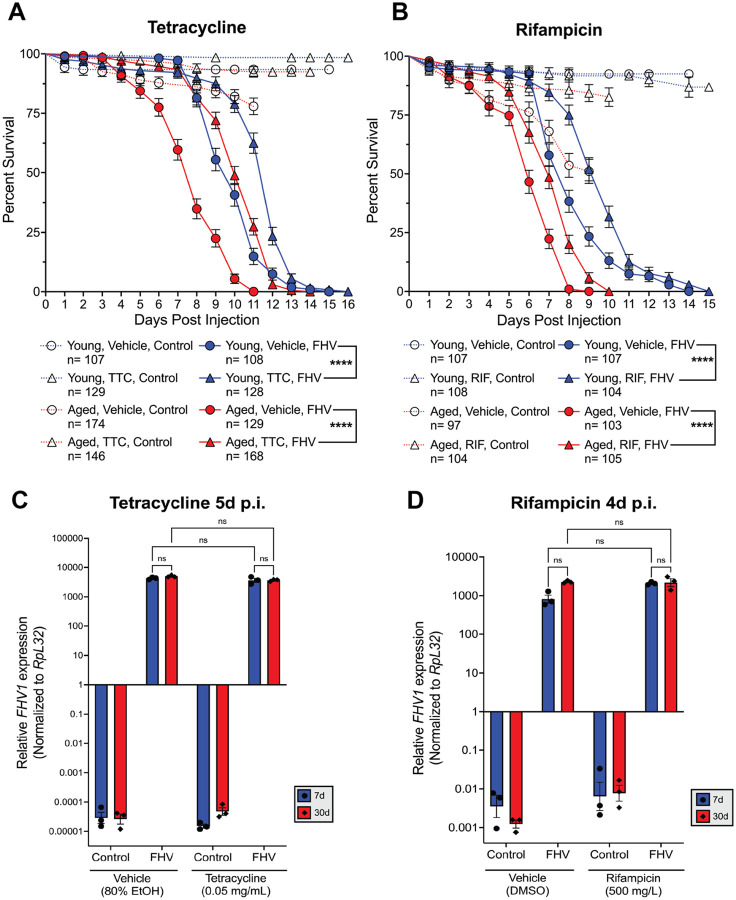
Tetracycline and rifampicin antibiotics extend survival after FHV infection without reducing virus load. (**A, B**) Survival curves of young and aged male *Oregon R* flies infected with FHV or control-injected with the same volume of cell culture media. The graphs compare the survival curves of young and aged males from 8 independent injection experiments (**A**) and 5 independent injection experiments (**B**). After injection, flies were then placed on TTC or vehicle food (**A**) and RIF or vehicle food (**B**). Statistics of FHV survival are based on a Log-Rank (Mantel-Cox) test. ****p<0.0001. (**C, D**) Virus load determined by *FHV1* expression reveals comparable titers between young and aged flies. Comparable titers were also observed between flies receiving the appropriate vehicle food and TTC (**C**) or RIF (**D**). Graphs represent mean +/− SEM from 3 biological replicates from groups of 5 flies. Statistics for virus load are based on two-way ANOVA followed by Tukey post-test to correct for multiple comparisons. ns= non-significant (*p*>0.05).

To determine how treatment with mitochondrial targeting antibiotics affected survival of FHV infection, we individually injected young and aged, wild-type *Oregon R* male flies with cell culture media (control) or FHV, then placed the flies on either vehicle- or antibiotic food recording the number of living flies each day. We found tetracycline (TTC) treatment significantly extends survival of young (*p* < 0.0001) and aged (*p* < 0.0001) flies after FHV infection (Fig. 1A). The median survival of young flies receiving vehicle food was 10 days. TTC treatment extended the median survival of young flies to 12 days, a 20% extension. The median survival of aged flies receiving vehicle food was 8 days. TTC treatment extended the median survival of aged flies to 10 days, a 25% extension. Aged flies fed TTC food (*p* = 0.1768) displayed the same median survival (10d) as young flies fed on vehicle food, demonstrating that TTC treatment of aged flies rescued the age-dependent susceptibility to FHV. Feeding flies on lower doses of TTC revealed a dose-dependent survival extension by TTC in young and aged flies (**Fig. S2**), as flies fed lower doses did not show extended survival. Rifampicin (RIF) treatment also significantly extended survival of young (*p* < 0.0001) and aged (*p* < 0.0001) flies (Fig. 1B). The median survival of young flies receiving vehicle food was 8 days. Rifampicin treatment extended the median survival of young flies to 10 days, a 25% extension. In aged flies, the median survival of flies receiving vehicle food was 6 days. Rifampicin treatment extended the median survival of aged flies to 7 days, a 16.7% extension.

Next, we sought to determine if the observed protection from each antibiotic treatment was a result of reducing virus load. We measured virus loads via RT-qPCR at 5d p.i. for TTC and 4d p.i. for RIF. At these respective timepoints, we began to observe differences in survival rates between young and aged flies fed the appropriate vehicle food. Conversely, survival rates between young and aged flies receiving TTC or RIF remained similar, demonstrating a protective effect of each antibiotic compared to its vehicle (Fig. 1A, B). For tetracycline’s vehicle (80% EtOH) at 5d p.i., the survival rate of young flies after infection was 100%, while aged flies’ survival rate was 84.5%. Young and aged flies receiving tetracycline had similar survival rates at this timepoint (93 and 97%, respectively). For rifampicin’s vehicle (DMSO) at 4d p.i., the survival rate of young flies after infection was 94.4%, while aged flies’ survival rate was 78.6%. Young and aged flies receiving rifampicin had similar survival rates at this timepoint (95.2 and 91.4%, respectively). We observed no significant difference in *FHV RNA1 (FHV1)* expression between flies receiving vehicle and TTC or RIF treatment, and independent of age (Fig. 1C, D). Together, these results show that TTC and RIF extend survival without reducing FHV load at timepoints where each antibiotic begins to demonstrate its protective effect. Therefore, it is likely that treatment with these antibiotics improves disease tolerance to FHV infection.

### Antimicrobial properties do not explain tetracycline-mediated survival extension

**Figure 2:**
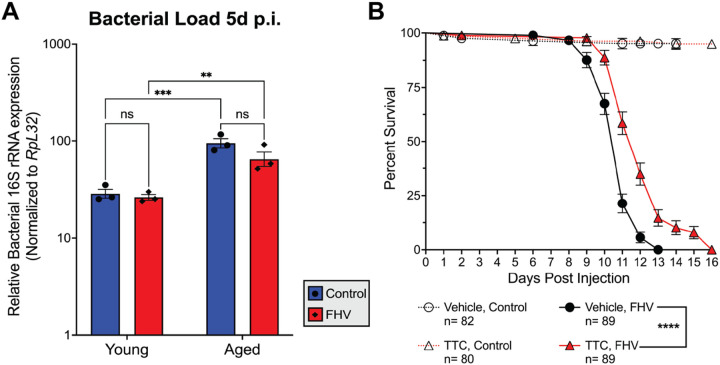
Survival extension by tetracycline is likely a result of non-antimicrobial properties. (**A**) Bacterial load 5d p.i. determined by bacterial *16S rRNA* copy number reveals comparable bacterial loads between control-injected and FHV-injected flies and significantly increased bacterial loads with age. Graphs represent +/− SEM of 3 biological replicates from groups of 10 flies. Statistics for bacterial load are based on two-way ANOVA followed by Tukey post-test to correct for multiple comparisons. ***p<0.001, **p<0.01, ns= non-significant (*p*>0.05). (**B**) Survival curves of axenic *Oregon R* males infected with FHV or control-injected with the same volume of cell-culture media. The graph compares the survival curves of axenic flies fed vehicle or TTC food from 5 independent injection experiments. ****p<0.0001.

To account for the possibility that antibiotic treatments may extend survival by mitigating secondary bacterial infections, we determined bacterial load by measuring the relative bacterial *16S rRNA* copy number [49]. We found that FHV infection did not significantly alter bacterial loads in young (*p* = 0.9567) or aged (*p* = 0.2025) flies (Fig. 2A), so the presence of secondary bacterial infections with FHV infection is not likely. Consistent with previous studies, we found a significant increase in bacterial loads with age regardless of treatment (Control: *p* = 0.0005, FHV: *p* = 0.0029) [52].

To address potential confounding effects of antibiotic treatment on the host microbiome, we generated axenic *Oregon R* males to assess their survival after FHV infection with vehicle- or TTC treatment. We found that TTC treatment extended the survival of FHV-infected axenic flies (*p* < 0.0001). The median survival of axenic flies receiving vehicle food was 11 days. TTC treatment extended the median survival of axenic flies to 12 days, a 9.1% extension. Our findings indicate that survival extension after TTC treatment occurs independently of potential alterations to the composition of the host’s microbiome.

#### Tetracycline treatment upregulates genes involved in the UPR ^mt^, glycolysis, and oxidative stress response after FHV infection

Due to its ability to induce mitochondrial stress, we hypothesized the protection with TTC treatment may result from expression changes of genes involved in UPR^mt^ activation, glucose metabolism, or the response to oxidative stress. Using RT-qPCR, we measured the expression of genes involved in the *Drosophila* UPR^mt^ [36, 53] or orthologs involved in the mammalian UPR^mt^ [54–56], glycolysis [57–68], and oxidative stress response at various timepoints (24h, 72h, and 120h p.i.). We analyzed genes involved in the oxidative stress response that we previously observed age-dependent differences in expression after FHV infection [14].

**Figure 3:**
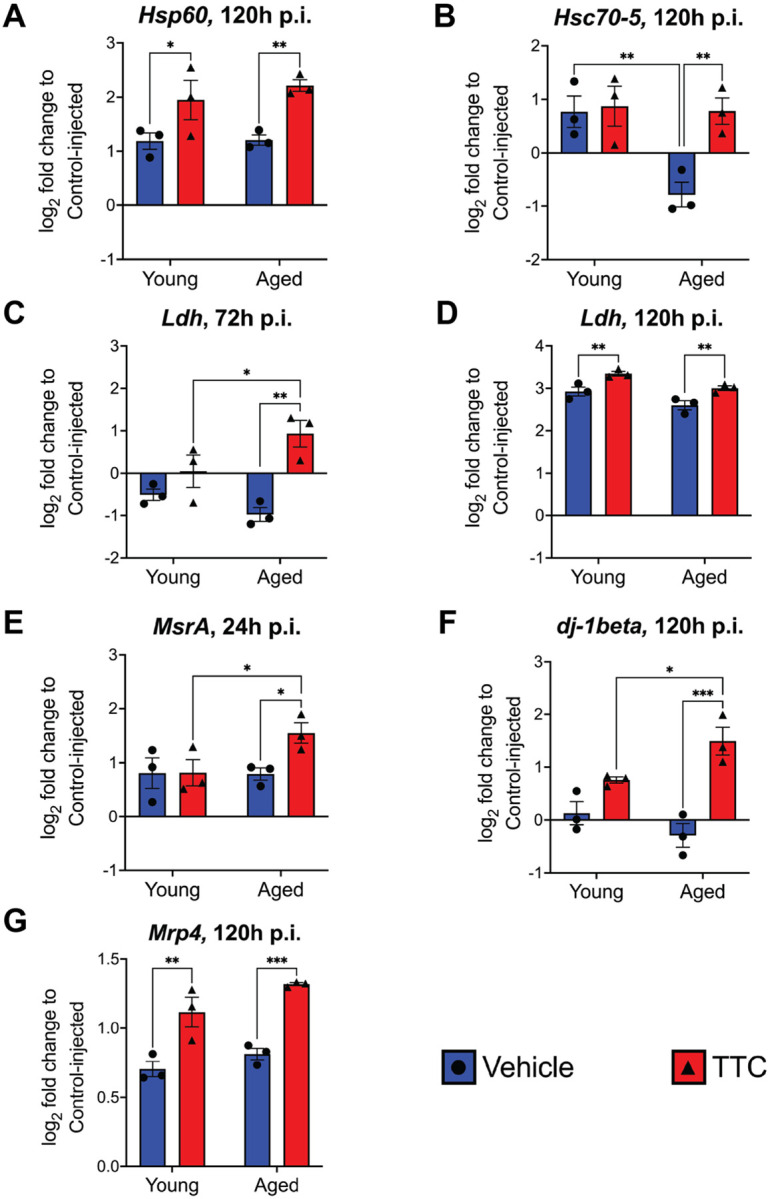
TTC treatment increased expression of genes involved in the UPR^mt^, glycolysis, and oxidative stress response after FHV infection. (**A-G**) Gene expression determined by RT-qPCR after FHV infection and comparing flies fed vehicle or TTC. Graphs depict +/− SEM of three biological replicates from groups of 5 flies. Statistics are based on a two-way ANOVA followed by Tukey post-test to correct for multiple comparisons. *p<0.05, **p<0.01, ***p<0.001.

We observed an increase in expression of genes involved in the UPR^mt^ after TTC treatment (Fig. 3A, B). We found that TTC treatment significantly increased *Hsp60* expression in both young and aged flies (*p* = 0.0343 and p = 0.0095, respectively) 120 hpi (Fig. 3A). We also observed an increase in *Hsc-70–5* expression with TTC treatment in aged flies 120 hpi (*p* = 0.0052). Although we did not observe relevant changes in gene expression with the other UPR^mt^ genes analyzed (Fig. S2), *Hsp60* and *Hsc-70–5* are UPR^mt^ markers encoding highly conserved mitochondrial chaperone proteins that function to restore mitochondrial homeostasis [56, 69]. These results demonstrate that UPR^mt^ activation occurs with TTC treatment after FHV infection, and its activation may be more pronounced in aged flies.

We observed increased expression of lactate dehydrogenase (*Ldh*), responsible for converting pyruvate to lactate and promoting glycolysis, after TTC treatment (Fig. 3C, D). At 72 hpi, this expression increase was specific to aged flies (*p* = 0.0010). At 120 hpi, we observed significantly increased *Ldh* expression with TTC treatment regardless of age (young: *p* = 0.0079, aged: *p* = 0.00393). This is consistent with previous studies that described increased expression of glycolysis genes with UPR^mt^ activation [70–72], indicating a metabolic shift from oxidative phosphorylation to glycolysis. We did not observe significant changes in expression of the remaining genes that we tested (Fig. S4), but this may be explained by the fact that many glycolytic enzymes are regulated post transcriptionally [73].

We also observed an increase in expression of genes involved in the oxidative stress response after TTC treatment (Fig. 3E–G). At 24 hpi, we observed increased expression of *MsrA* (*p* = 0.0.379) in aged flies (Fig. 3E). At 120 hpi, *dj-1beta* and *Mrp4* expression was significantly increased after TTC treatment (Fig. 3F, G). A significant increase in expression of *dj-1beta* was observed in aged flies with TTC treatment; young flies also showed increased expression with treatment, but this was not statistically significant. *Mrp4* expression significantly increased regardless of age with TTC treatment (young: *p* = 0.0019, aged: *p* = 0.0005). We did not observe significant changes in expression of the remaining genes we tested (Fig. S5). These findings suggest that TTC treatment improved the oxidative stress response.

##### ND23 ^60114^ mutants display longer end-point survival of FHV compared to wild type controls

**Figure 4:**
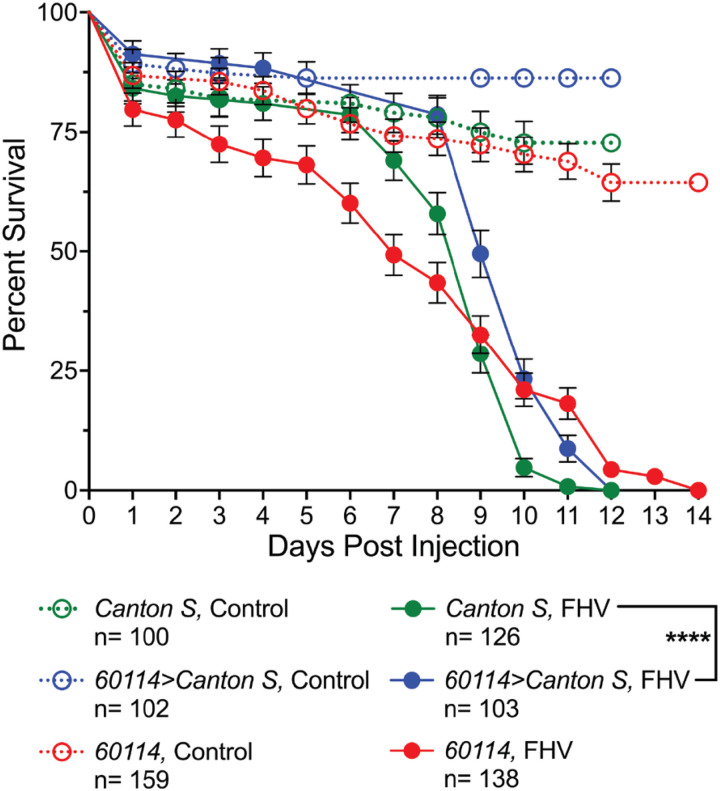
*ND23*^*60114*^ mutants are protected following FHV infection. Survival curves of *Canton S*, *ND23*^*60114*^, and *ND23*^*60114*^>*Canton S* heterozygous flies infected with FHV or control-injected with the same volume of cell culture media. The graphs compare the survival curves from at least 3 independent injection experiments. ****p<0.0001.

As an additional approach, we sought to determine whether mitochondrial stress resulting from a genetic mutation could also improve survival outcomes after FHV infection. To do so, we monitored survival of FHV-infected *ND23*^*60114*^ homozygous mutants relative to *wild type (WT)* Canton S and Canton *S/ND23*^*60114*^ *(ND23*^*60114*^*/+)* heterozygotes. We found no significant differences in FHV survival of *ND23*^*60114*^ homozygous mutants compared to *Canton S* (*p* = 0.2490) or *ND23*^*60114*^*/+* heterozygotes (*p* = 0.1117). However, we did find that the *ND23*^*60114*^*/+* heterozygotes survived FHV infection significantly better than *Canton S* flies (*p* < 0.0001, Fig. 4). This result suggests that one mutated copy of the *ND23* gene could potentially induce a milder mitochondrial stress resulting in a hormetic effect. This survival improvement aligns with previous work showing *ND23*^*60114*^*/+* heterozygotes had significantly increased lifespan compared to *Canton S* [34]. Although the median survival of *ND23*^*60114*^ homozygotes was shorter (7d)

compared to *Canton S* and heterozygotes (both 9d), *ND23*^*60114*^ mutants had a later end-point survival. At 11d, the survival rate of *ND23*^*60114*^ mutants was 18.1%, while *Canton S* and heterozygote survival rates were 0.8% and 8.7%, respectively. It is worth noting that male *ND23*^*60114*^ mutants exhibit a significantly shorter lifespan in comparison to *Canton S* and heterozygous *ND23*^*60114*^*/+* flies (~ 20d vs 50d and 60d, respectively [34]), suggesting that the later time point extension of FHV survival in the homozygous mutant is significant.

## Discussion

In this study, we tested whether inducing mitochondrial dysfunction could extend survival in young and aged *Drosophila* after FHV infection. We found that treatment with mitochondrial targeting antibiotics extended survival in *Drosophila* melanogaster regardless of age. We observed greater protection of FHV infection with TTC compared to RIF (young: TTC = 12d vs RIF = 10d, aged: TTC = 10d vs RIF = 7d). This could be a result of the different mechanisms of action of these antibiotics or simply that the doses of antibiotics tested did not induce an equivalent level of stress. Therefore, it appears that the type of mitochondrial stressor or intensity of stress could influence the strength of the adaptive response and impact survival outcomes after infection. This notion was further supported by the observed dose-dependent survival improvement of TTC, as we found that lower doses reduced the survival extension or did not provide protection (**Fig. S2**). Interestingly, TTC provided better protection of FHV to aged flies, as we observed a 25% median survival increase compared to 20% in young flies. Conversely, RIF provided better protection to young flies, resulting in a 25% survival increase compared to 16.7% in aged flies. Future experiments could compare the effects of both antibiotics on mitochondrial function using high-resolution respirometry.

We showed that TTC and RIF extend survival without reducing FHV load, indicating improved disease tolerance is likely responsible for the observed protection. A recent study reported that rifampicin has antiviral effects in cultured S2 cells infected with two other RNA viruses, significantly reducing virus load [33]. However, this was observed after DCV and CrPV infection, which are both picorna-like viruses, unlike FHV which is a nodavirus. Haas et al. pretreated the cells for 24h with increasing RIF doses of 0, 25, 50, and 100 μM. Meanwhile, in this study we only tested RIF at 500 mg/L or 607.6 μM. FHV has been shown to significantly downregulate *Cyp6a8* expression in aged flies at 24h and 48 p.i.[14], which is one of the genes that Haas et al. reported to be upregulated after RIF treatment [33]. While we did not observe significant changes in FHV load after RIF treatment, we cannot exclude the possibility that feeding the antibiotic to FHV-infected flies results in changes of *Cyp6a8* expression and is associated with the observed improved outcomes. Further studies are needed to confirm this *in vivo* and in the context of FHV infection. Future studies should also investigate whether rifampicin has an antiviral effect *in vivo* following infection with viruses such as DCV and CrPV. UPR^mt^ activation after TTC treatment has been shown to improve disease tolerance mechanisms in mice models of bacterial sepsis and influenza virus infection [24, 25]. In the context of sepsis, the lungs and liver showed decreased tissue damage with TTC treatment, which was associated with distinct tissue-specific transcriptional signatures that improved survival by promoting tissue repair or reprogramming metabolism [24]. Likewise, the improved tolerance after influenza virus infection was associated with induction of genes involved in lung epithelial cell and cilia function, as well as downregulated inflammatory and immune genes in the lungs, liver, and kidneys to reduce immunopathology [25]. Based on our findings and existing literature, we could investigate tissue-specific disease tolerance mechanisms (reviewed in [74]) in future studies to elucidate potential mechanisms that improve survival. Tissue-specific transcriptomic analyses of tissues with known FHV tropism could identify genes implicated in disease tolerance to FHV induced by TTC or RIF.

We concluded that the survival extension of FHV was not likely a result of TTC’s antimicrobial properties. We found bacterial loads did not significantly increase with FHV infection, which suggests antimicrobial properties of the antibiotics were not mitigating potential secondary bacterial infections. We also showed survival extension in TTC-treated axenic flies exposed to FHV, so TTC’s survival extension was not likely a result of altering the host’s microbiome. We recognize that assessing bacterial load by quantifying bacterial *16S rRNA* copy number is an approach with limitations. Therefore, future studies could aim to characterize the host microbiome with *16S rRNA* gene amplicon sequencing, allowing us to characterize microbial composition and relative abundance. Alternatively, we could repeat similar experiments with 9-tert-butyl doxycycline, a derivative of TTC shown to have minimal antimicrobial properties but maintained ability to induce the UPR^mt^ [25]. These approaches could allow us to conclude more convincingly that TTC’s protection is not a result of antimicrobial properties.

Since TTC treatment extended survival better in aged flies relative to young, we sought to determine if there were differences in expression of genes involved in the UPR^mt^, glycolytic metabolism, and oxidative stress response following TTC treatment. *Hsp60* and *Hsc-70–5* are evolutionarily conserved across *C. elegans*, mammals, and flies as mitochondrial chaperone proteins involved in the UPR^mt^, and are considered one of the best assessments of UPR^mt^ activation [42, 75]. We found increased expression of these UPR^mt^ markers with TTC treatment. We observed increased expression of *Ldh* after TTC treatment. This increase in *Ldh* expression is also correlated with our expected reduction of OCR with TTC treatment, as it indicates a metabolic shift from oxidative phosphorylation to glycolysis. This metabolic shift has been shown to be involved in UPR^mt^ activation. Together, these results suggest that TTC treatment activates the UPR^mt^, perhaps with better induction in aged flies.

We observed increases in expression in oxidative stress response genes with TTC treatment. This is consistent with previous reports of increased expression of oxidative stress response genes with UPR^mt^ activation [72]. The gene products of *dj-1beta* and *MsrA* are highly conserved antioxidants [76, 77]. In response to paraquat-induced oxidative stress, *Mrp4* was shown to be necessary and sufficient for transcription of JNK-dependent antioxidant genes. The increased expression that we observed of these genes suggests an improved oxidative stress response after TTC treatment limits oxidative damage and promotes cell survival, offering another potential mechanism to extend survival.

We demonstrated that *ND23*^*60114*^ mutants had a longer end-point survival to FHV compared to *wildtype* flies, despite having a significantly shorter lifespan. Alternatively, we could knockdown *ND23* or *ND75* ubiquitously using the Gal4/UAS system combined with the Gal80^ts^ factor to induce knockdown in adults, as ubiquitous knockdown is lethal when induced in development [78]. This approach could resolve the difficulties of making comparisons between mutants and controls with such significant lifespan differences and perhaps provide more convincing conclusions.

Our results confirmed the hypothesis that interventions reducing metabolic rates, or inducing hypometabolism, serve as a disease tolerance mechanism that improves survival outcomes after FHV infection. However, we do not completely understand the mechanisms by which this survival extension occurs. Is this simply a result of UPR^mt^ activating protective stress responses that allow cellular survival? Is this a result of a metabolic shift that serves to reduce mitochondrial stress and promote function? Since the *Drosophila* UPR^mt^ remains relatively uncharacterized compared to the UPR^mt^ of *C. elegans* and mammals, it may be difficult to elucidate the mechanisms that are conferring protection at this point. For example, different mitochondrial stressors have been shown to activate different transcription factors in *C. elegans* and mammals, implicating multiple branches of the UPR^mt^ (reviewed in [79]). Various experimental models have described UPR^mt^ activation to be protective in disease states or extend longevity, which has made it a popular target for therapeutics. While the evidence is considerable, there are negative consequences that come with prolonged UPR^mt^ activation [80–82]. Additionally, *C. elegans* lifespan studies have shown that the timing of UPR^mt^ activation can affect its protective effect. UPR^mt^ activation during development resulted in lifespan extension; however, induction after adulthood did not extend lifespan [83]. Further research is necessary to better understand which mitochondrial stressors are activating specific branches of the UPR^mt^. However, many studies suggest that activating cellular and mitochondrial stress responses could be a valuable therapeutic target in many different contexts.

## Supplementary Material

Supplementary Files

This is a list of supplementary files associated with this preprint. Click to download.

- SupplementalMaterial.docx

## References

[1] Rajan A and Perrimon N. Drosophila as a Model for Interorgan Communication: Lessons from Studies on Energy Homeostasis. Developmental Cell. 2011; 21(1):29–31. 21763605 10.1016/j.devcel.2011.06.034 PMC3560414

[2] Davies KJ. Adaptive homeostasis. Mol Aspects Med. 2016; 49:1–7. 27112802 10.1016/j.mam.2016.04.007 PMC4868097

[3] Pomatto LCD and Davies KJA. The role of declining adaptive homeostasis in ageing. J Physiol. 2017; 595(24):7275–7309. 29028112 10.1113/JP275072 PMC5730851

[4] Walford RL. THE IMMUNOLOGIC THEORY OF AGING. Immunological Reviews. 1969; 2(1):171–171.

[5] Nikolich-Zugich J, Knox KS, Rios CT, Natt B, Bhattacharya D and Fain MJ. SARS-CoV-2 and COVID-19 in older adults: what we may expect regarding pathogenesis, immune responses, and outcomes. Geroscience. 2020; 42(2):505–514. 32274617 10.1007/s11357-020-00186-0 PMC7145538

[6] Montgomery RR. Age-related alterations in immune responses to West Nile virus infection. Clin Exp Immunol. 2017; 187(1):26–34. 27612657 10.1111/cei.12863 PMC5167051

[7] Hernandez-Vargas EA, Wilk E, Canini L, Toapanta FR, Binder SC, Uvarovskii A, Ross TM, Guzmán CA, Perelson AS and Meyer-Hermann M. Effects of aging on influenza virus infection dynamics. J Virol. 2014; 88(8):4123–4131. 24478442 10.1128/JVI.03644-13 PMC3993746

[8] Leng J and Goldstein DR. Impact of aging on viral infections. Microbes and Infection. 2010; 12(14):1120–1124. 20849976 10.1016/j.micinf.2010.08.009 PMC2998572

[9] Economic UNDo and Affairs S. (2023). World Social Report 2023: United Nations).

[10] Venter PA and Schneemann A. Recent insights into the biology and biomedical applications of Flock House virus. Cell Mol Life Sci. 2008; 65(17):2675–2687. 18516498 10.1007/s00018-008-8037-y PMC2536769

[11] Merkling SH, Bronkhorst AW, Kramer JM, Overheul GJ, Schenck A and Van Rij RP. The epigenetic regulator G9a mediates tolerance to RNA virus infection in Drosophila. PLoS Pathog. 2015; 11(4):e1004692. 25880195 10.1371/journal.ppat.1004692 PMC4399909

[12] Eleftherianos I, Won S, Chtarbanova S, Squiban B, Ocorr K, Bodmer R, Beutler B, Hoffmann JA and Imler JL. ATP-sensitive potassium channel (K(ATP))-dependent regulation of cardiotropic viral infections. Proc Natl Acad Sci U S A. 2011; 108(29):12024–12029. 21719711 10.1073/pnas.1108926108 PMC3141999

[13] Ertel KJ, Benefield D, Castaño-Diez D, Pennington JG, Horswill M, den Boon JA, Otegui MS and Ahlquist P. Cryo-electron tomography reveals novel features of a viral RNA replication compartment. eLife. 2017; 6:e25940. 28653620 10.7554/eLife.25940 PMC5515581

[14] Sheffield L, Sciambra N, Evans A, Hagedorn E, Goltz C, Delfeld M, Kuhns H, Fierst JL and Chtarbanova S. Age-dependent impairment of disease tolerance is associated with a robust transcriptional response following RNA virus infection in Drosophila. G3 Genes|Genomes|Genetics. 2021; 11(7). 10.1093/g3journal/jkab116 PMC8495950 33836060

[15] Galiana-Arnoux D, Dostert C, Schneemann A, Hoffmann JA and Imler JL. Essential function in vivo for Dicer-2 in host defense against RNA viruses in drosophila. Nat Immunol. 2006; 7(6):590–597. 16554838 10.1038/ni1335

[16] Thomson TC, Schneemann A and Johnson J. Oocyte destruction is activated during viral infection. genesis. 2012; 50(6):453–465. 22173880 10.1002/dvg.22004

[17] Liu B, Behura SK, Clem RJ, Schneemann A, Becnel J, Severson DW and Zhou L. P53-mediated rapid induction of apoptosis conveys resistance to viral infection in Drosophila melanogaster. PLoS Pathog. 2013; 9(2):e1003137. 23408884 10.1371/journal.ppat.1003137 PMC3567152

[18] Lamiable O, Arnold J, de Faria I, Olmo RP, Bergami F, Meignin C, Hoffmann JA, Marques JT and Imler JL. Analysis of the Contribution of Hemocytes and Autophagy to Drosophila Antiviral Immunity. J Virol. 2016; 90(11):5415–5426. 27009948 10.1128/JVI.00238-16 PMC4934735

[19] Hagedorn E, Bunnell D, Henschel B, Smith DL, Dickinson S, Brown AW, De Luca M, Turner AN and Chtarbanova S. RNA virus-mediated changes in organismal oxygen consumption rate in young and old Drosophila melanogaster males. Aging (Albany NY). 2023; 15(6):1748–1767. 36947702 10.18632/aging.204593 PMC10085608

[20] Gorr TA. Hypometabolism as the ultimate defence in stress response: how the comparative approach helps understanding of medically relevant questions. Acta Physiologica. 2017; 219(2):409–440. 27364602 10.1111/apha.12747

[21] Storey KB and Storey JM. Metabolic rate depression in animals: transcriptional and translational controls. Biological Reviews. 2004; 79(1):207–233. 15005178 10.1017/s1464793103006195

[22] Ganeshan K, Nikkanen J, Man K, Leong YA, Sogawa Y, Maschek JA, Van Ry T, Chagwedera DN, Cox JE and Chawla A. Energetic Trade-Offs and Hypometabolic States Promote Disease Tolerance. Cell. 2019; 177(2):399–413.e312. 30853215 10.1016/j.cell.2019.01.050 PMC6456449

[23] Booser DJ and Hortobagyi GN. Anthracycline antibiotics in cancer therapy. Focus on drug resistance. Drugs. 1994; 47(2):223–258. 7512899 10.2165/00003495-199447020-00002

[24] Colaço HG, Barros A, Neves-Costa A, Seixas E, Pedroso D, Velho T, Willmann KL, Faisca P, Grabmann G, Yi HS, Shong M, Benes V, Weis S, Tetracycline Antibiotics Induce Host-Dependent Disease Tolerance to Infection. Immunity. 2021; 54(1):53–67.e57. 33058782 10.1016/j.immuni.2020.09.011 PMC7840524

[25] Mottis A, Li TY, El Alam G, Rapin A, Katsyuba E, Liaskos D, D’Amico D, Harris NL, Grier MC, Mouchiroud L, Nelson ML and Auwerx J. Tetracycline-induced mitohormesis mediates disease tolerance against influenza. J Clin Invest. 2022; 132(17). 10.1172/JCI151540 PMC9433105 35787521

[26] Tauber SC and Nau R. Immunomodulatory properties of antibiotics. Current molecular pharmacology. 2008; 1(1):68–79. 20021425

[27] Pradhan S, Madke B, Kabra P and Singh AL. Anti-inflammatory and Immunomodulatory Effects of Antibiotics and Their Use in Dermatology. Indian J Dermatol. 2016; 61(5):469–481. 27688434 10.4103/0019-5154.190105 PMC5029230

[28] Singh R, Sripada L and Singh R. Side effects of antibiotics during bacterial infection: Mitochondria, the main target in host cell. Mitochondrion. 2014; 16:50–54. 24246912 10.1016/j.mito.2013.10.005

[29] Moullan N, Mouchiroud L, Wang X, Ryu D, Williams EG, Mottis A, Jovaisaite V, Frochaux MV, Quiros PM, Deplancke B, Houtkooper RH and Auwerx J. Tetracyclines Disturb Mitochondrial Function across Eukaryotic Models: A Call for Caution in Biomedical Research. Cell Rep. 2015; 10(10):1681–1691. 25772356 10.1016/j.celrep.2015.02.034 PMC4565776

[30] Chopra I and Roberts M. Tetracycline antibiotics: mode of action, applications, molecular biology, and epidemiology of bacterial resistance. Microbiol Mol Biol Rev. 2001; 65(2):232–260; second page, table of contents. 11381101 10.1128/MMBR.65.2.232-260.2001 PMC99026

[31] Wehrli W. Rifampin: mechanisms of action and resistance. Rev Infect Dis. 1983; 5 Suppl 3:S407–411. 6356275 10.1093/clinids/5.supplement_3.s407

[32] Goldenthal M and Nishiura JT. Isolation and characterization of a mitochondrial RNA polymerase from Drosophila melanogaster. Biochem Cell Biol. 1987; 65(2):173–182. 3103654 10.1139/o87-022

[33] Haas G, Seiler M, Nguyen J, Troxler L, Pennarun S, Lefebvre E, Benamrouche Y, Loizeau L, Reinbolt C, Liang M, Lin X, Li W, Xia Z, Regulation of detoxifying enzymes expression and restriction of picorna-like virus infection by natural polysaccharide extracts in Drosophila cells. Virology. 2025; 607:110513. 40163969 10.1016/j.virol.2025.110513

[34] Loewen CA and Ganetzky B. Mito-Nuclear Interactions Affecting Lifespan and Neurodegeneration in a Drosophila Model of Leigh Syndrome. Genetics. 2018; 208(4):1535–1552. 29496745 10.1534/genetics.118.300818 PMC5887147

[35] Olufs ZPG, Wassarman DA and Perouansky M. Stress Pathways Induced by Volatile Anesthetics and Failure of Preconditioning in a Mitochondrial Complex I Mutant. Anesthesiology. 2024; 140(3):463–482. 38118175 10.1097/ALN.0000000000004874 PMC10932926

[36] Morrow G, Le Pécheur M and Tanguay RM. Drosophila melanogaster mitochondrial Hsp22: a role in resistance to oxidative stress, aging and the mitochondrial unfolding protein response. Biogerontology. 2016; 17(1):61–70. 26155908 10.1007/s10522-015-9591-y

[37] Liu X, Jiang N, Hughes B, Bigras E, Shoubridge E and Hekimi S. Evolutionary conservation of the clk1-dependent mechanism of longevity: loss of mclk1 increases cellular fitness and lifespan in mice. Genes Dev. 2005; 19(20):2424–2434. 16195414 10.1101/gad.1352905 PMC1257397

[38] Copeland JM, Cho J, Lo T Jr., Hur JH, Bahadorani S, Arabyan T, Rabie J, Soh J and Walker DW. Extension of Drosophila life span by RNAi of the mitochondrial respiratory chain. Curr Biol. 2009; 19(19):1591–1598. 19747824 10.1016/j.cub.2009.08.016

[39] Rea SL, Ventura N and Johnson TE. Relationship between mitochondrial electron transport chain dysfunction, development, and life extension in Caenorhabditis elegans. PLoS Biol. 2007; 5(10):e259. 17914900 10.1371/journal.pbio.0050259 PMC1994989

[40] Martinus RD, Garth GP, Webster TL, Cartwright P, Naylor DJ, Høj PB and Hoogenraad NJ. Selective Induction of Mitochondrial Chaperones in Response to Loss of the Mitochondrial Genome. European Journal of Biochemistry. 1996; 240(1):98–103. 8797841 10.1111/j.1432-1033.1996.0098h.x

[41] Zhao Q, Wang J, Levichkin IV, Stasinopoulos S, Ryan MT and Hoogenraad NJ. A mitochondrial specific stress response in mammalian cells. Embo j. 2002; 21(17):4411–4419. 12198143 10.1093/emboj/cdf445 PMC126185

[42] Yoneda T, Benedetti C, Urano F, Clark SG, Harding HP and Ron D. Compartment-specific perturbation of protein handling activates genes encoding mitochondrial chaperones. J Cell Sci. 2004; 117(Pt 18):4055–4066. 15280428 10.1242/jcs.01275

[43] Durieux J, Wolff S and Dillin A. The cell-non-autonomous nature of electron transport chain-mediated longevity. Cell. 2011; 144(1):79–91. 21215371 10.1016/j.cell.2010.12.016 PMC3062502

[44] Suárez-Rivero JM, Pastor-Maldonado CJ, Povea-Cabello S, Álvarez-Córdoba M, Villalón-García I, Talaverón-Rey M, Suárez-Carrillo A, Munuera-Cabeza M, Reche-López D, Cilleros-Holgado P, Piñero-Pérez R and Sánchez-Alcázar JA. Activation of the Mitochondrial Unfolded Protein Response: A New Therapeutic Target? Biomedicines. 2022; 10(7). 10.3390/biomedicines10071611 PMC9313171 35884915

[45] Owusu-Ansah E, Song W and Perrimon N. Muscle Mitohormesis Promotes Longevity via Systemic Repression of Insulin Signaling. Cell. 2013; 155(3):699–712. 24243023 10.1016/j.cell.2013.09.021 PMC3856681

[46] Pickering AM, Vojtovich L, Tower J and KJ AD. Oxidative stress adaptation with acute, chronic, and repeated stress. Free Radic Biol Med. 2013; 55:109–118. 23142766 10.1016/j.freeradbiomed.2012.11.001 PMC3687790

[47] Merkling SH and van Rij RP. Analysis of resistance and tolerance to virus infection in Drosophila. Nature Protocols. 2015; 10(7):1084–1097. 26110714 10.1038/nprot.2015.071

[48] Oh CT, Moon C, Choi TH, Kim BS and Jang J. Mycobacterium marinum infection in Drosophila melanogaster for antimycobacterial activity assessment. J Antimicrob Chemother. 2013; 68(3):601–609. 23118147 10.1093/jac/dks425

[49] Pais IS, Valente RS, Sporniak M and Teixeira L. Drosophila melanogaster establishes a species-specific mutualistic interaction with stable gut-colonizing bacteria. PLOS Biology. 2018; 16(7):e2005710. 29975680 10.1371/journal.pbio.2005710 PMC6049943

[50] Gloor GB, Preston CR, Johnson-Schlitz DM, Nassif NA, Phillis RW, Benz WK, Robertson HM and Engels WR. Type I repressors of P element mobility. Genetics. 1993; 135(1):81–95. 8224830 10.1093/genetics/135.1.81 PMC1205629

[51] Marchesi JR, Sato T, Weightman AJ, Martin TA, Fry JC, Hiom SJ, Dymock D and Wade WG. Design and evaluation of useful bacterium-specific PCR primers that amplify genes coding for bacterial 16S rRNA. Appl Environ Microbiol. 1998; 64(2):795–799. 9464425 10.1128/aem.64.2.795-799.1998 PMC106123

[52] Ren C, Webster P, Finkel SE and Tower J. Increased internal and external bacterial load during Drosophila aging without life-span trade-off. Cell Metab. 2007; 6(2):144–152. 17681150 10.1016/j.cmet.2007.06.006

[53] Baqri RM, Pietron AV, Gokhale RH, Turner BA, Kaguni LS, Shingleton AW, Kunes S and Miller KE. Mitochondrial chaperone TRAP1 activates the mitochondrial UPR and extends healthspan in Drosophila. Mechanisms of Ageing and Development. 2014; 141–142:35–45. 10.1016/j.mad.2014.09.002 PMC4310785 25265088

[54] Wang G, Fan Y, Cao P and Tan K. Insight into the mitochondrial unfolded protein response and cancer: opportunities and challenges. Cell & Bioscience. 2022; 12(1):18. 10.1186/s13578-022-00747-0 PMC8857832 35180892

[55] Runkel ED, Baumeister R and Schulze E. Mitochondrial stress: balancing friend and foe. Exp Gerontol. 2014; 56:194–201. 24603155 10.1016/j.exger.2014.02.013

[56] Bennett CF, Vander Wende H, Simko M, Klum S, Barfield S, Choi H, Pineda VV and Kaeberlein M. Activation of the mitochondrial unfolded protein response does not predict longevity in Caenorhabditis elegans. Nature Communications. 2014; 5(1):3483. 10.1038/ncomms4483 PMC3984390 24662282

[57] Slaninova V, Krafcikova M, Perez-Gomez R, Steffal P, Trantirek L, Bray SJ and Krejci A. Notch stimulates growth by direct regulation of genes involved in the control of glycolysis and the tricarboxylic acid cycle. Open Biol. 2016; 6(2):150155. 26887408 10.1098/rsob.150155 PMC4772804

[58] Szablewski L. Expression of glucose transporters in cancers. Biochim Biophys Acta. 2013; 1835(2):164–169. 23266512 10.1016/j.bbcan.2012.12.004

[59] Christofk HR, Vander Heiden MG, Harris MH, Ramanathan A, Gerszten RE, Wei R, Fleming MD, Schreiber SL and Cantley LC. The M2 splice isoform of pyruvate kinase is important for cancer metabolism and tumour growth. Nature. 2008; 452(7184):230–233. 18337823 10.1038/nature06734

[60] Desai S, Ding M, Wang B, Lu Z, Zhao Q, Shaw K, Yung WK, Weinstein JN, Tan M and Yao J. Tissue-specific isoform switch and DNA hypomethylation of the pyruvate kinase PKM gene in human cancers. Oncotarget. 2014; 5(18):8202–8210. 24077665 10.18632/oncotarget.1159 PMC4226677

[61] Schell JC, Olson KA, Jiang L, Hawkins AJ, Van Vranken JG, Xie J, Egnatchik RA, Earl EG, DeBerardinis RJ and Rutter J. A role for the mitochondrial pyruvate carrier as a repressor of the Warburg effect and colon cancer cell growth. Mol Cell. 2014; 56(3):400–413. 25458841 10.1016/j.molcel.2014.09.026 PMC4268416

[62] Bricker DK, Taylor EB, Schell JC, Orsak T, Boutron A, Chen YC, Cox JE, Cardon CM, Van Vranken JG, Dephoure N, Redin C, Boudina S, Gygi SP, A mitochondrial pyruvate carrier required for pyruvate uptake in yeast, Drosophila, and humans. Science. 2012; 337(6090):96–100. 22628558 10.1126/science.1218099 PMC3690818

[63] Baek G, Tse YF, Hu Z, Cox D, Buboltz N, McCue P, Yeo CJ, White MA, DeBerardinis RJ, Knudsen ES and Witkiewicz AK. MCT4 defines a glycolytic subtype of pancreatic cancer with poor prognosis and unique metabolic dependencies. Cell Rep. 2014; 9(6):2233–2249. 25497091 10.1016/j.celrep.2014.11.025

[64] Hitosugi T, Fan J, Chung TW, Lythgoe K, Wang X, Xie J, Ge Q, Gu TL, Polakiewicz RD, Roesel JL, Chen GZ, Boggon TJ, Lonial S, Tyrosine phosphorylation of mitochondrial pyruvate dehydrogenase kinase 1 is important for cancer metabolism. Mol Cell. 2011; 44(6):864–877. 22195962 10.1016/j.molcel.2011.10.015 PMC3246218

[65] Kaplon J, Zheng L, Meissl K, Chaneton B, Selivanov VA, Mackay G, van der Burg SH, Verdegaal EM, Cascante M, Shlomi T, Gottlieb E and Peeper DS. A key role for mitochondrial gatekeeper pyruvate dehydrogenase in oncogene-induced senescence. Nature. 2013; 498(7452):109–112. 23685455 10.1038/nature12154

[66] Miao P, Sheng S, Sun X, Liu J and Huang G. Lactate dehydrogenase A in cancer: a promising target for diagnosis and therapy. IUBMB Life. 2013; 65(11):904–910. 24265197 10.1002/iub.1216

[67] Eichenlaub T, Villadsen R, Freitas FCP, Andrejeva D, Aldana BI, Nguyen HT, Petersen OW, Gorodkin J, Herranz H and Cohen SM. Warburg Effect Metabolism Drives Neoplasia in a Drosophila Genetic Model of Epithelial Cancer. Curr Biol. 2018; 28(20):3220–3228.e3226. 30293715 10.1016/j.cub.2018.08.035

[68] Gándara L, Durrieu L, Behrensen C and Wappner P. A genetic toolkit for the analysis of metabolic changes in Drosophila provides new insights into metabolic responses to stress and malignant transformation. Scientific Reports. 2019; 9(1):19945. 31882718 10.1038/s41598-019-56446-3 PMC6934733

[69] Pareek G, Thomas RE, Vincow ES, Morris DR and Pallanck LJ. Lon protease inactivation in Drosophila causes unfolded protein stress and inhibition of mitochondrial translation. Cell Death Discovery. 2018; 4(1):51. 10.1038/s41420-018-0110-1 PMC6197249 30374414

[70] Nargund AM, Fiorese CJ, Pellegrino MW, Deng P and Haynes CM. Mitochondrial and nuclear accumulation of the transcription factor ATFS-1 promotes OXPHOS recovery during the UPR(mt). Mol Cell. 2015; 58(1):123–133. 25773600 10.1016/j.molcel.2015.02.008 PMC4385436

[71] Mouchiroud L, Houtkooper RH, Moullan N, Katsyuba E, Ryu D, Cantó C, Mottis A, Jo YS, Viswanathan M, Schoonjans K, Guarente L and Auwerx J. The NAD(+)/Sirtuin Pathway Modulates Longevity through Activation of Mitochondrial UPR and FOXO Signaling. Cell. 2013; 154(2):430–441. 23870130 10.1016/j.cell.2013.06.016 PMC3753670

[72] Nargund AM, Pellegrino MW, Fiorese CJ, Baker BM and Haynes CM. Mitochondrial import efficiency of ATFS-1 regulates mitochondrial UPR activation. Science. 2012; 337(6094):587–590. 22700657 10.1126/science.1223560 PMC3518298

[73] Ni X, Lu C-p, Xu G-q and Ma J-j. Transcriptional regulation and post-translational modifications in the glycolytic pathway for targeted cancer therapy. Acta Pharmacologica Sinica. 2024; 45(8):1533–1555. 38622288 10.1038/s41401-024-01264-1 PMC11272797

[74] Medzhitov R, Schneider DS and Soares MP. Disease Tolerance as a Defense Strategy. Science. 2012; 335(6071):936–941. 22363001 10.1126/science.1214935 PMC3564547

[75] Houtkooper RH, Mouchiroud L, Ryu D, Moullan N, Katsyuba E, Knott G, Williams RW and Auwerx J. Mitonuclear protein imbalance as a conserved longevity mechanism. Nature. 2013; 497(7450):451–457. 23698443 10.1038/nature12188 PMC3663447

[76] Meulener MC, Xu K, Thomson L, Ischiropoulos H and Bonini NM. Mutational analysis of DJ-1 in Drosophila implicates functional inactivation by oxidative damage and aging. Proc Natl Acad Sci U S A. 2006; 103(33):12517–12522. 16894167 10.1073/pnas.0601891103 PMC1533799

[77] Chung H, Kim AK, Jung SA, Kim SW, Yu K and Lee JH. The Drosophila homolog of methionine sulfoxide reductase A extends lifespan and increases nuclear localization of FOXO. FEBS Lett. 2010; 584(16):3609–3614. 20655917 10.1016/j.febslet.2010.07.033

[78] Zeng X, Han L, Singh SR, Liu H, Neumüller RA, Yan D, Hu Y, Liu Y, Liu W, Lin X and Hou SX. Genome-wide RNAi screen identifies networks involved in intestinal stem cell regulation in Drosophila. Cell Rep. 2015; 10(7):1226–1238. 25704823 10.1016/j.celrep.2015.01.051 PMC4420031

[79] Shpilka T and Haynes CM. The mitochondrial UPR: mechanisms, physiological functions and implications in ageing. Nature Reviews Molecular Cell Biology. 2018; 19(2):109–120. 29165426 10.1038/nrm.2017.110

[80] Martinez BA, Petersen DA, Gaeta AL, Stanley SP, Caldwell GA and Caldwell KA. Dysregulation of the Mitochondrial Unfolded Protein Response Induces Non-Apoptotic Dopaminergic Neurodegeneration in C. elegans Models of Parkinson’s Disease. J Neurosci. 2017; 37(46):11085–11100. 29030433 10.1523/JNEUROSCI.1294-17.2017 PMC5688521

[81] Angeli S, Foulger A, Chamoli M, Peiris TH, Gerencser A, Shahmirzadi AA, Andersen J and Lithgow G. The mitochondrial permeability transition pore activates the mitochondrial unfolded protein response and promotes aging. Elife. 2021; 10. 10.7554/eLife.63453 PMC8410078 34467850

[82] O’Malley J, Kumar R, Inigo J, Yadava N and Chandra D. Mitochondrial Stress Response and Cancer. Trends in Cancer. 2020; 6(8):688–701. 32451306 10.1016/j.trecan.2020.04.009 PMC7392807

[83] Rea SL, Ventura N and Johnson TE. Relationship Between Mitochondrial Electron Transport Chain Dysfunction, Development, and Life Extension in Caenorhabditis elegans. PLOS Biology. 2007; 5(10):e259. 17914900 10.1371/journal.pbio.0050259 PMC1994989

